# Kidney weight and volume among living donors in Brazil

**DOI:** 10.1590/S1516-31802007000400006

**Published:** 2007-07-01

**Authors:** Vladimir Tonello De Vasconcelos, Rafael Cauê Katayama, Maria Flavia de Lima Ribeiro, José Osmar Medina-Pestana, José Carlos Costa Baptista-Silva

**Keywords:** Living donor, Allograft, Kidney, Kidney transplantation, Organ size, Doadores vivos, Transplante homólogo, Rim, Transplante de rim, Peso do órgão

## Abstract

**CONTEXT AND OBJECTIVE:**

The present study was performed to measure kidney weight and volume among living donors of both sexes in Brazil.

**DESIGN AND SETTING:**

This was a cross-sectional survey carried out between December 2001 and August 2004.

**METHODS:**

Kidney transplantations from 219 living donors were analyzed for this study. The kidneys were weighed in grams on a single-pan digital balance just after drainage of the perfusion fluid and removal of the perirenal fat. The kidney volume was determined in milliliters by water displacement.

**RESULTS:**

The mean age at nephroureterectomy was 44 ± 9.5. The donor organs came from the left side in 172 cases and from the right side in 47 cases. The weights and volumes of the right and left kidneys were, respectively, 169.83 ± 29.91 g and 157.38 ± 31.84 ml; and 173.00 ± 33.52 g and 160.34 ± 34.40 ml. The differences between the sides were not significant.

**CONCLUSIONS:**

According to the present study, kidney weight cannot be the only factor determining the side on which nephroureterectomy is performed, because of the lack of statistical significance between the two sides. On average, females donate lower nephron doses than males do, which could in some transplants result in allograft damage.

## RESUMO

**CONTEXTO E OBJETIVO:**

O atual estudo foi realizado para medir massa e volume de rins de doadores vivos brasileiros de ambos os sexos.

**TIPO DE ESTUDO E LOCAL:**

Estudo transversal executado entre dezembro de 2001 e agosto de 2004, foram incluídos no estudo, 219 nefroureterectomias renais de doadores vivos.

**MÉTODOS:**

O rim do doador foi pesado em gramas em uma balança digital, imediatamente após a retirada da gordura peri-renal e drenagem do fluído perfusional. O volume renal foi determinado em mililitros por deslocamento de água.

**RESULTADOS:**

A média de idade dos doadores foi de 44 ± 9,5 anos. Foram realizadas 172 (78,54%) nefroureterectomias no lado esquerdo e 47 (21,46%) no lado direito. O peso renal do lado direito foi de 169,83 ± 29,91 g; enquanto que o peso renal do lado esquerdo foi de 173,00 ± 33,52 g. O volume renal do lado direito foi de 157,38 ± 31,84 ml; do lado esquerdo, 160,34 ± 34,40 ml. Não havendo diferença significativa entre os lados.

**CONCLUSÕES:**

De acordo com o estudo atual, não há diferença significativa entre o peso renal do lado direito e esquerdo, não podendo ser o peso renal um fator de escolha do lado da nefroureterectomia. Além disso, o estudo proporciona uma sobre o peso e volume de rins de brasileiros adultos de ambos os sexos.

## INTRODUCTION

Some studies on kidney weight have been carried out in certain Western countries and also in India^1^ and Korea.^2^ The Indian study was done using cadaveric kidneys obtained from medicolegal autopsies, while the Korean study presented data based on kidneys from living organ donors.^1,2^ It is well known that kidney transplantation from living donors presents a better outcome than do cadaveric kidney allografts.^3,4^ In addition, grafted kidney function in kidney transplants from living donors is limited by the relative graft functional mass.^5,6^

## OBJECTIVE

The present study was performed to measure kidney weights and volumes among living donors of both sexes in Brazil. Correlations between kidney weight and volume and body weight were investigated. However, it is important to mention that racial stratification was considered to be inappropriate in this study because of the intense miscegenation of the Brazilian population.

## METHODS

This was a cross-sectional study carried out between December 2001 and August 2004, in which 219 kidney transplantations from living donors that were performed by one of the present authors (JCCBS) were enrolled. Patients with incomplete data or graft loss were excluded from the study.

Before the nephroureterectomies, all the patients were cleared through conventional preoperative living donor protocols for transplantation. At this point, weight, height, age, gender, relationship between the donor and recipient, blood type and cross-match compatibility were recorded. The immunological evaluation included cytotoxic and flow cytometric cross-matching, and also human leukocyte antigen (HLA) class I and II typing. Renal vasculature and anatomy were evaluated preoperatively by aortography or magnetic resonance imaging (MRI) angiogram. Routine preanesthesia tests (complete blood count, multiple serum analysis, urinalysis and coagulation studies) were also performed at this time.

All the nephroureterectomies were performed by means of an anterior retroperitoneal approach and the incision size was recorded. The kidneys were weighed in grams on a single-pan digital balance (Filizola BP6), just after drainage of the perfusion fluid and removal of the perirenal fat. The kidney volume was determined in milliliters by means of water displacement.

The other variables studied included transplantation history, anastomosis, duration of the operation and volume of blood lost during surgery. All these data were recorded for future studies on long-term patient and graft survival following kidney transplantations.

The data were analyzed using the Statistical Package for the Social Sciences (SPSS) software, version 11.0. Pearson's correlation coefficient was calculated to assess the relationships between kidney volume and body weight and between those parameters and other variables. Single factor analysis of variance (Anova) was used to evaluate differences in mean values among groups. All p-values were two-tailed and p < 0.05 was considered significant.

This project was approved by the Ethics Committee of Universidade Federal de São Paulo.

## RESULTS

The overall number of female donors in our series of 219 nephroureterectomies was 137 (63%). The donor organs came from the left side in 172 cases (78.54%), and from the right side in 47 cases. The patients’ mean age at the time of nephroureterectomy was 44 ± 9.5 (range: 23 to 72). Their mean weight was 74.49 ± 11.63 kg for males and 65.64 ± 11.43 kg for females. The mean height was 1.68 ± 0.06 m for males and 1.58 ± 0.08 m for females. The donor's body mass index (BMI) ranged from 16.8 kg/m^2^ to 39.8 kg/m^2^ (mean: 26.2 kg/m^2^).

The mean kidney weight and volume were 172.32 ± 32.74 g and 159.70 ± 33.82 ml, respectively. Although the kidney weights and volumes for the two genders were not statistically different between the four age groups (Table 1), this might be explained by the low number of cases in the first age group. On the other hand, the kidney weights and volumes were significantly correlated (p < 0.01) with body weights and BMI (Table 2). There were no significant differences in relation to which side the kidney was from, for any of the parameters (Table 3).

**Table 1.** Means and standard deviations of kidney weights and volumes according to age groups

Age group (years)	n	Kidney weight (g)	Kidney volume (ml)
**20-30**	13	158.23 ± 32.79	148.92 ± 28.98
**31-40**	70	170.66 ± 31.90	157.44 ± 33.51
**41-50**	88	173.76 ± 31.48	160.88 ± 33.11
**51**	48	175.92 ± 35.95	163.77 ± 36.79
**Anova**		p = 0.344	p = 0.490

Anova = analysis of variance.

**Table 2.** Correlations (p-values) between kidney weight, kidney volume, donor weight and body mass index (BMI)

	Kidney weight	Kidney volume
**Donor weight**	0.587	0.523
**Kidney volume**	0.94	------
**BMI**	0.451	0.414

Correlation is significant at the 0.01 level (two-tailed). BMI = kg/m^2^.

**Table 3.** Means, standard deviations (SD), and ranges of various kidney and patient measurements according to side

	Side	n	Mean	SD	Minimum	Maximum	P
**Kidney weight (g)**	Left	172	173.00	33.52	95.00	262.00	0.558
	Right	47	169.83	29.91	108.00	240.00	
	Total	219	172.32	32.74	95.00	262.00	
**Kidney volume (ml)**	Left	172	160.34	34.40	58.00	254.00	0.597
	Right	47	157.38	31.84	95.00	228.00	
	Total	219	159.70	33.82	58.00	254.00	
**Donor weight (kg)**	Left	172	69.09	12.07	46.00	103.00	0.752
	Right	47	68.45	13.03	40.00	106.00	
	Total	219	68.95	12.25	40.00	106.00	
**Donor height (m)**	Left	172	1.62	0.09	1.40	1.89	0.357
	Right	47	1.61	0.09	1.44	1.78	
	Total	219	1.62	0.09	1.40	1.89	
**Body mass index (BMI)**	Left	172	26.18	3.91	16.81	39.84	0.812
	Right	47	26.34	4.25	16.87	38.95	
	Total	219	26.22	3.98	16.81	39.84	

The mean weights of the right and left kidneys were, respectively, 177.43 ± 23.88 g and 187.07 ± 34.69 g for males and 166.61 ± 31.92 g and 163.80 ± 29.44 g for females. A similar pattern was evident for the volumes (Table 4). Except in relation to BMI, right kidney weight and volume, gender differences were significant for all other measurements (Table 4). Also, the differences between the sides in relation to kidney weight, kidney volume, donor weight, donor height and BMI were not significant for either gender (Table 4).

**Table 4.** Means and standard deviations (SD) of various kidney and patient measurements according to gender

		Male (M)		Female		
	Side	n	Mean ± SD	n	Mean ± SD	p (M/F)
**Kidney weight (g)**	Right (R)	14	177.43 ± 23.88	33	166.61 ± 31.92	0.261
	Left (L)	68	187.07 ± 34.69	104	163.80 ± 29.43	< 0.001
	Both	82	185.43 ± 33.17	137	164.47 ± 29.96	< 0.001
	p (R/L)		0.179		0.262	
**Kidney volume (ml)**	R	14	165.07 ± 30.64	33	154.12 ± 32.24	0.286
	L	68	174.90 ± 35.70	104	150.82 ± 30.07	< 0.001
	Both	82	173.22 ± 34.91	137	151.61 ± 30.52	< 0.001
	p (R/L)		0.592		0.488	
**Donor weight (kg)**	R	14	74.86 ± 13.92	33	65.73 ± 11.82	0.026
	L	68	74.41 ± 11.21	104	65.61 ± 11.36	< 0.001
	Both	82	74.49 ± 11.63	137	65.64 ± 11.43	< 0.001
	p (R/L)		0.741		0.899	
**Donor height (m)**	R	14	1.70 ± 0.05	33	1.57 ± 0.08	< 0.001
	L	68	1.68 ± 0.07	104	1.59 ± 0.08	< 0.001
	Both	82	1.68 ± 0.06	137	1.58 ± 0.08	< 0.001
	p (R/L)		0.36		0.635	
**BMI (kg/m2)**	R	14	25.95 ± 3.98	33	26.51 ± 4.40	0.682
	L	68	26.39 ± 3.84	104	26.05 ± 3.97	0.571
	Both	82	26.32 ± 3.85	137	26.16 ± 4.07	0.775
	p (R/L)		0.579		0.852	

BMI = body mass index = kg/m^2^; M = male; F = female.

## DISCUSSION

At birth, both kidneys weigh between 20 and 35 g. The average adult kidney weight is 10 to 14 times greater than the newborn kidney weight.^7^ Nyengaard and Bendtsen^8^ (1992) showed that there is a linear relationship between kidney weight and total glomerular count.

From the present study, it can be seen that kidney weight cannot be the only factor determining the side on which to perform nephroureterectomy, because of the lack of statistical difference between the two sides.

The discrepancy between kidney weights found by several studies (Table 5) can be explained by the different weights and heights of the populations and by the measurement methods. On fixation, kidney weight decreases by up to 10%. According to Saxena et al.,^9^ kidney volume measurements by means of ultrasonography have limitations because volumes are derived indirectly from two-dimensional measurements. In the present study, intraoperative weight and volume displacement measurements were made in bloodless kidneys, thereby providing more accurate parameters.

**Table 5.** Comparison between the present findings for kidney weight and volume and those in other studies

Authors (year)	Gender	Side	n	Weight (g)	Volume (ml)
**Sahni et al.^1^ (2001)**	Male	Right	155	108.7	
		Left	155	111.8	
	Female	Right	84	96.6	
		Left	84	99.4	
**Saxena et al.^9^ (2004)**	Male		26		199 ± 32
	Female		30		154 ± 27
**Pourmand et al.^5^ (2001)**			79	164	
**Kim et al.^2^ (1998)**			259	220.7 ± 39.9	
**Present study**	Male	Right	14	177.43 ± 23.88	165.07 ± 30.64
		Left	68	187.07 ± 34.69	174.90 ± 35.70
	Female	Right	33	166.61 ± 31.92	154.12 ± 32.24
		Left	104	163.80 ± 29.43	150.82 ± 30.07

It has been suggested that inadequacy of transplanted kidney mass relative to recipient size may be a non-immunological factor that leads to graft loss.^10^

## CONCLUSION

As seen in this study, indirect measurements show that, on average, females donate lower nephron doses than males do, which could in some transplants result in allograft damage. Kidney weight cannot be the only factor determining the side on which nephroureterectomy is performed, because of the lack of statistical significance between the two sides. Moreover, the present study provides baseline data on the weights and volumes of the kidneys of Brazilian adults of both sexes. In addition, the data recorded at the time of transplantation may provide information on the role of glomerular filtration in cases of chronic renal allograft failure.
